# Reduction in the incidence of pneumonia in elderly patients after hip fracture surgery: An inpatient pulmonary rehabilitation program

**DOI:** 10.1097/MD.0000000000011845

**Published:** 2018-08-17

**Authors:** Shih-Chieh Chang, Jiun-I Lai, Mei-Chin Lu, Kuan-Hung Lin, Wei-Shu Wang, Su-Shun Lo, Yi-Chun Lai

**Affiliations:** a School of Medicine; b Institute of Hospital and Health Care Administration; c Institute of Public Health, National Yang-Ming University, Taipei; d Division of Chest Medicine; e Division of Endocrine; f Department of Medicine, Division of Oncology; g Department of Critical Care Medicine; h Department of Surgery, National Yang-Ming University Hospital, Yilan, Taiwan, Republic of China.

**Keywords:** elderly, pneumonia, postoperative, pulmonary rehabilitation

## Abstract

Hip fracture is an important health care issue in the elderly. Postoperative pulmonary complications occur in 4% of patients after hip fracture surgery. However, previous research is limited regarding pulmonary rehabilitation in this group. In this study, we present clinical evidence regarding the impact of a comprehensive pulmonary rehabilitation program in elderly hip fracture patients after hip surgery.

We designed a nonrandomized, Quasi-experimental study, comparing 2 sequential time periods in the same center. Elderly patients (≥65 years) with a new hip fracture from February 1, 2014 to December 31, 2015 and who were willing to undergo a postoperative pulmonary rehabilitation program were enrolled. The pulmonary rehabilitation program started on January 1, 2015. Patients who refused rehabilitation or did not receive a surgical intervention were excluded. Patients received either standard care (standard care group) or standard care plus the postoperative rehabilitation program (intervention group).

A total of 240 patients (163 women and 77 men) were enrolled, including 138 in the standard care group and 102 in the intervention group. The intervention group had a significantly lower incidence of pneumonia (6 patients, 5.9%) compared to the standard care group (19 patients, 13.9%). An age >80 years, cancer status, and not undergoing the postoperative pulmonary rehabilitation program were factors associated with a higher risk of pneumonia. In multivariate analysis, age >80 years, history of stroke/cancer, thrombocytopenia, and hyperglycemia (>200 mg/dL) were identified as risk factors for pneumonia.

The incidence of pneumonia was lower in the elderly patients with hip fractures who received the postoperative pulmonary rehabilitation program after surgery. This is the first trial to demonstrate the effect of a postoperative pulmonary rehabilitation program in hip surgery patients.

## 1 Introduction

The incidence of hip fracture in the elderly has risen rapidly from 1.7 million in 1990 to an estimated 6.3 million in 2050 worldwide.^[1]^ Disability, defined as loss or impairment of baseline daily performance after the fracture event, has been reported in two-thirds of this group of patients.^[2]^ After surgery for hip fracture repair, higher rates of serious complications from pulmonary disorders such as pneumonia, atelectasis, and pulmonary thromboembolism have been reported in patients older than 60 years.^[3]^ Once serious pulmonary complications develop, the 30-day mortality rate has been reported to be as high as 17%.^[3]^

The role of pulmonary rehabilitation in reducing postoperative pulmonary complications in cardiothoracic and abdominal surgery has been established.^[4]^ Methods of pulmonary rehabilitation include lung expansion modalities such as incentive spirometry, deep-breathing exercises, chest physiotherapy, and cough-assisted maneuvers. These risk reduction strategies have been studied in patients with postoperative atelectasis, pneumonia and hypoxemia, and have been proven to be effective.^[5]^ Strong predictors of mortality after hip fracture surgery include advanced age, male sex, nursing home residence, poor preoperative walking capacity, poor activities of daily living, higher ASA (American Society of Anesthesiologists) grade, multiple comorbidities, dementia or cognitive impairment, diabetes, cancer, and cardiac disease.^[6]^

However, no previous study has investigated the role of pulmonary rehabilitation focusing on elderly patients after hip surgery. Therefore, we conducted an integrated hip fracture and osteoporosis rehabilitation program (I-HOPE program) in our hospital targeting this demographic, and evaluated whether postoperative pulmonary complications were reduced by this program.

## 2 Methods

We designed a nonrandomized, Quasi-experimental study, comparing 2 sequential time periods in the same center. In this study, we organized a multidisciplinary team including orthopedists, physiatrists, physical therapists, respiratory therapists, nursing staff and nutritionists. The integrated hip fracture and osteoporosis rehabilitation program of the elderly (I-HOPE program) was initiated on February 1, 2014 following approval of the Institutional Review Board (IRB) of National Yang-Ming University Hospital in Yi-Lan (IRB approved No 2016A013). The inclusion criteria were: age ≥ 65 years; newly developed hip fracture; and willingness to undergo postoperative pulmonary rehabilitation. The exclusion criteria were: refusal to undergo the postoperative pulmonary rehabilitation program; multiple bone fractures; no required surgical intervention; and coexisting hip fracture and brain injury. The baseline period started from February 1, 2014 to December 31, 2014. The pulmonary rehabilitation program started from January 1, 2015 to December 31, 2015

**Figure 1:**
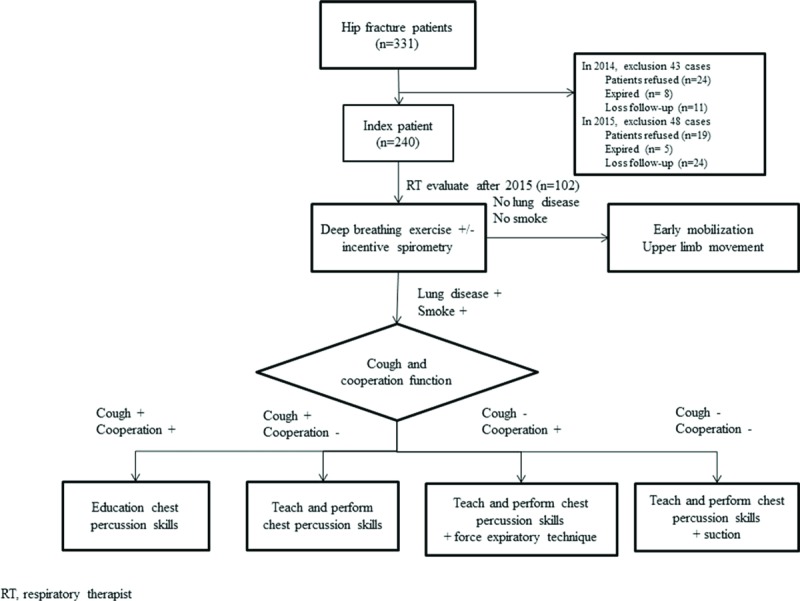
The flow chart of the procedures of the inpatient pulmonary rehabilitation program for each patient.

The postoperative inpatient pulmonary rehabilitation program was initiated immediately on the day following surgery, and included deep breathing exercises/incentive spirometry, chest physiotherapy, and cough-assisted maneuvers such as oscillatory techniques^[3]^ and finished till the patient discharged. The flowchart of the procedures used for the inpatient pulmonary rehabilitation program is presented in Figure 1. We arranged deep breathing exercises, incentive spirometry, chest percussion therapy, and forced expiratory techniques in all of the enrolled patients. The pulmonary rehabilitation program was stopped immediately at any time if the patient reported discomfort.

The standard care group received usual medical treatment and care by the physician in charge (Fig. 1), and enrolled 181 patients from February 6, 2014 to December 31, 2104. We excluded 43 cases (24 who refused to participate, 8 who died before collection, and 11 who were lost to follow-up), and the remaining 128 patients were included in the analysis. The intervention group (standard care plus pulmonary rehabilitation) received the postoperative inpatient pulmonary rehabilitation program and standard medical treatment care, and enrolled 150 patients from January 5, 2015 to November 27, 2015. We excluded 48 patients (19 who refused to participate, and 5 who died before collection). Both groups were followed up at 1 month, 3 months, and 12 months after hip fracture surgery.

A diagnosis of pneumonia was defined as the use of antibiotics plus postoperative chest radiographs consistent with new infiltrates and compatible clinical symptoms such as fever, cough, sputum, among others, identified by the physician. Clinical parameters including age, sex, comorbidities, laboratory data, type of surgery, date of surgery, and date of a diagnosis of pneumonia were collected and analyzed. The consumption of antibiotics is represented by defined daily doses (DDD) per 1000 patient-days.^[7]^

### 2.1 Statistical analysis

All statistical analyses were conducted using SPSS software for Windows (version 22; IBM Corporation, Armonk, NY). Data are presented as frequencies for categorical variables and means ± standard deviations for numerical variables. Categorical variables were compared using the *χ*^2^ test or Fisher exact test, and continuous variables were compared using an independent unpaired *t* test. Univariate analysis was performed to evaluate the characteristics of postoperative pneumonia. Multivariate analysis was performed to evaluate the independent risk factor of postoperative pneumonia under logistic regression*. P* values of <0.05 were considered to be statistically significant.

## 3 Results

A total of 240 patients (163 women and 77 men) including 138 in the standard care group and 102 in the intervention group were enrolled. There were no significant differences in age, sex, comorbidities, laboratory data, and type of surgery between the standard care and intervention groups (Table 1). The length of hospital stay was significantly shorter in the intervention group compared with the standard care group (6.3 ± 1.8 days vs. 7.2 ± 3.6 days, *P* = .022).

**Table 1:**
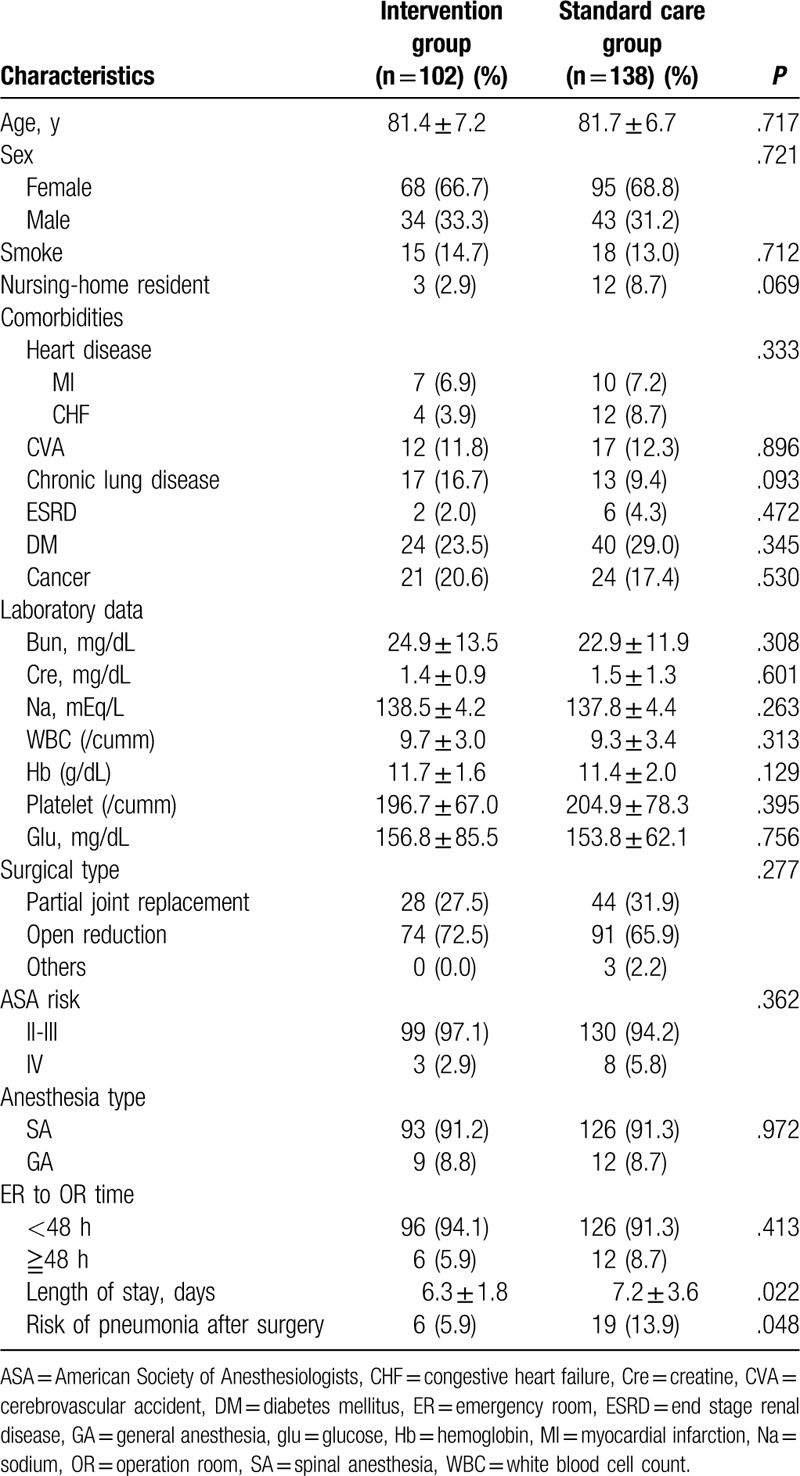
The characteristics of the standard care group and intensive care group.

**Figure 2:**
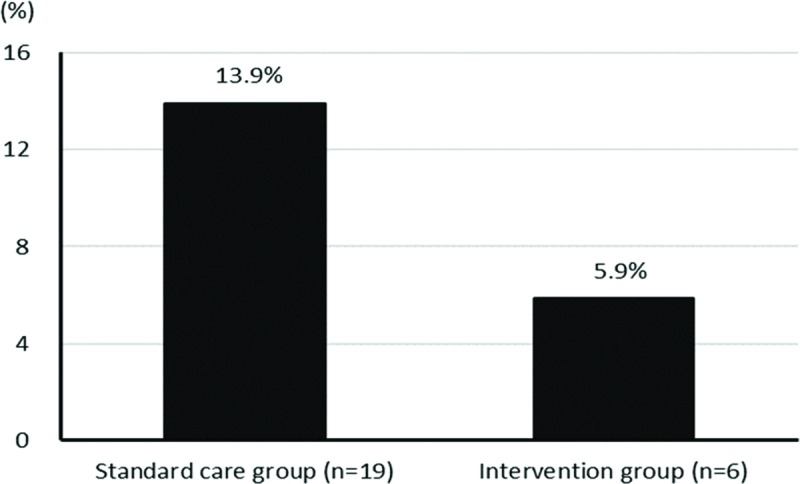
The percentage of postoperative pneumonia in the standard care group was 13.9% with a total of 19 patients. The percentage of postoperative pneumonia in the intervention group was 5.9% with a total of 6 patients.

In the standard care group, 19 patients (13.9%) developed pneumonia, compared to 6 patients (5.9%) in the intervention group (Table 2, Fig. 2). An age >80 years (*P* = .038), cancer status (*P* = .029), and not receiving the postoperative pulmonary rehabilitation program (*P* = .048) were associated with a higher risk of pneumonia (Table 2).

**Table 2:**
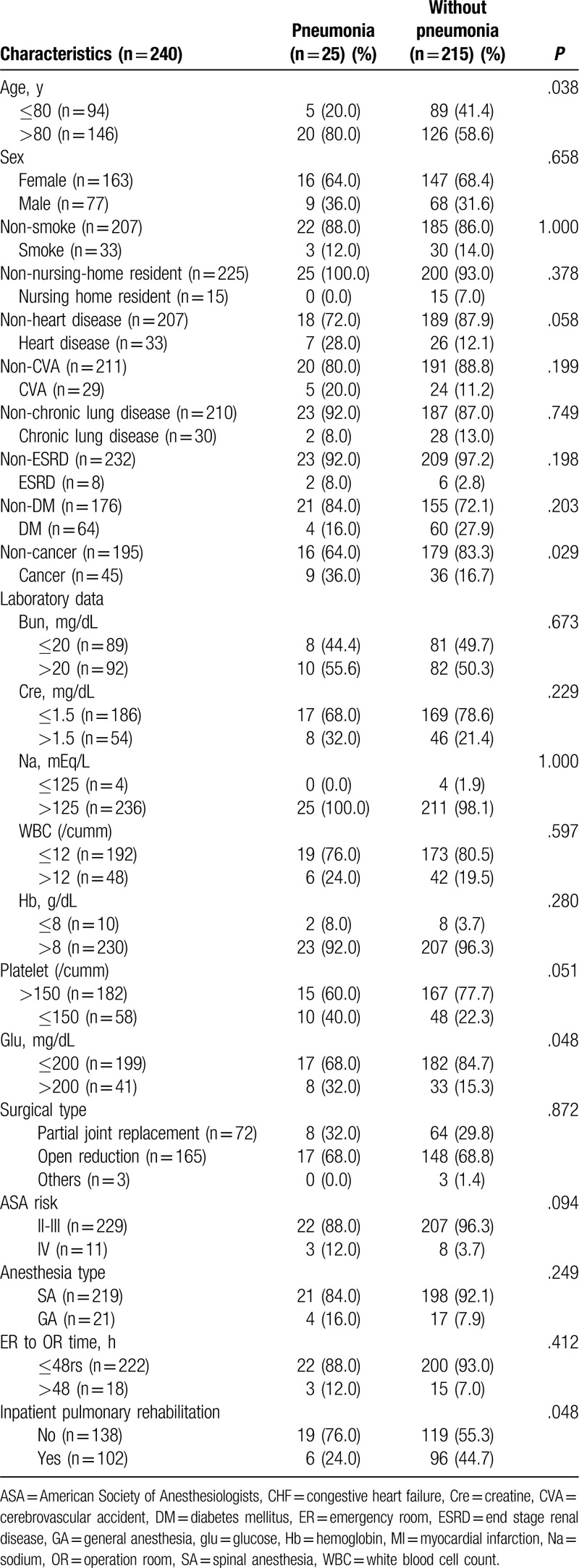
The characteristics of postoperative pneumonia.

In the multivariate analysis (Table 3), old age (odds ratio [OR] = 4.4, *P* = .016), presence of a cerebrovascular accident (OR = 4.5, *P* = .019), cancer (OR = 4.1, *P* = .006), thrombocytopenia (OR = 2.9, *P* = .041), and hyperglycemia (OR = 24.8, *P* < .001) were associated with a higher probability of pneumonia after hip fracture repair.

**Table 3:**
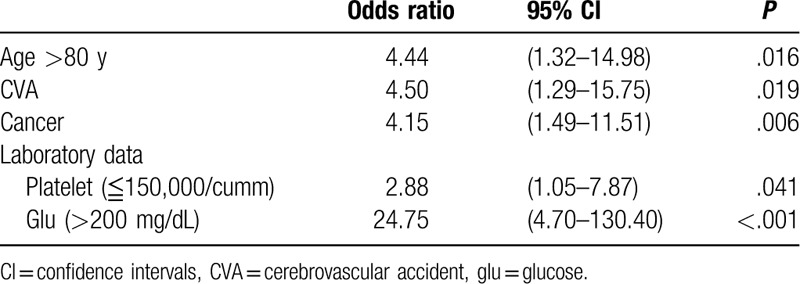
The multivariate of risk for postoperative pneumonia.

About the overall antibiotic consumption in the 2 groups, we found the intervention group had lower DDDs per 1000 patient-days than standard care group (82.3 vs. 232.4, *P* = .02). The 1-year all-cause mortality is 5.9% in the intervention group compared with the standard case group 7.2% (*P* = .796).

## 4 Discussion

In Taiwan, the proportion of people older than 65 years increased from 8.3% in 1998 to 12.0% in 2014,^[8]^ including 13.08% in Yilan in 2014.^[8]^ The mean age of the patients with hip fractures in this study was 81 years, and most of them suffered from fractures because of a falling accident, which is a common cause of hip fractures in the elderly. Etiologies of hip fractures include osteoporosis, which occurs more frequently in women, and an increased risk of osteoporotic fractures in postmenopausal women has been well documented. Medical illnesses including diabetes mellitus, malignancy, and cerebrovascular accidents were the most common underlying comorbidities in our patients, and they could have also contributed to falling accidents.^[9]^ The pneumonia patients with diabetes were only 4 cases (total 25 pneumonia patients). The 4 patients had higher blood sugar (>200 mg/dL) during admission than other diabetes without pneumonia. However, the case number is too small to make the conclusion. Therefore, if the diabetes patient had high blood pressure (>200 mg/dL) during admission, the hyperglycemia could be a potential risk factor for postoperative pneumonia after hip surgery. Low platelet count was an independent risk factor for postoperative pneumonia in our study. Bronheim et al^[10]^ also reported that low platelet count was an independent risk factor after posterior lumbar fusion for organ space surgical site infections (OR = 6.0, P < 0.001). Another study revealed declining platelet count predicts poor outcome for community-acquired pneumonia patients.^[11]^

During the first year of the I-HOPE program, a high incidence of pneumonia (13.9%) after surgical repair of hip fracture was noted. Moreover, compared to the patients without pneumonia, those with postoperative pneumonia had a higher in-hospital mortality rate (33.3% vs. 4.2%). Similarly, Lawrence et al^[3]^ also reported a 44% 1-year mortality rate once serious pulmonary complications developed after hip fracture repair. Another study^[12]^ focusing on nursing home residents with post-hip fracture complications including pneumonia and pressure ulcers within 6 months reported an even higher mortality rate of >70%. The high mortality rates in the literature suggest that effective strategies to prevent complications and facilitate recovery are essential.

Changes in pulmonary function owing to respiratory muscle functional impairment are common in patients receiving cardiac, thoracic, and upper abdominal surgery.^[13]^ Such changes can lead to lung atelectasis, hypoxemia, decreased cough function, and infection as postoperative pulmonary complications. A comprehensive pulmonary rehabilitation program has been shown to be effective in reducing postoperative pulmonary complications, especially in high-risk surgical patients such as those with chronic lung disease, cardiothoracic surgery, and abdominal surgery.^[14]^

Although detailed data on changes in pulmonary function after hip surgery are currently lacking, the spectrum of pulmonary complications are similar to those seen in cardiothoracic and other high risk surgeries. Common complications include atelectasis, pneumonia, respiratory failure, acute respiratory distress syndrome, and others.^[15]^ This suggests that the mechanism responsible for postoperative pulmonary complications may be similar regardless of the type of surgery. It may therefore be beneficial to apply previous postoperative pulmonary care strategies^[14,16]^ that have shown good efficacy in preventing complications to hip fracture surgery.

After the initiation of the I-HOPE program, the incidence of postoperative pneumonia fell to 5.9%. The clinical benefits of chest physiotherapy were not significant in the patients with a length of hospital stay of <1 week. During the subacute period (1–3 months after surgery), the incidence of pneumonia was significantly reduced in the pulmonary rehabilitation group (4.35% vs. 0%, *P* = .04).

In previous studies,^[12]^ complications of pulmonary infection after hip fracture have mainly occurred within 3 months postoperatively, although some cases have occurred after 3 to 6 months. Therefore, compared to standard care, combined pulmonary rehabilitation can also shorten the length of hospital stay and minimize health care costs. The microorganisms associated with pneumonia after hip fracture surgery are similar to those in hospital-acquired pneumonia. The treatment strategy for this subtype of postoperative pneumonia can be established after definite culture results have been obtained.

There are several limitations to this study, including the nonrandomized design, relatively small sample size, and lack of lung physiology measurements such as pulmonary function, arterial blood gas, and lung volume reduction on chest x-ray. However, to the best of our knowledge, this is the first study to analyze the impact of an inpatient pulmonary rehabilitation program on postoperative pulmonary complications in elderly patients after hip fracture surgery. Our results suggest the benefits of establishing a multidisciplinary team to improve the quality of care in this clinical setting.

In conclusion, multidisciplinary care including a pulmonary rehabilitation team can shorten the hospitalization course and may reduce the incidence of pneumonia during the recovery period after discharge.

## Author contributions

**Data curation:** Yi-Chun Lai, Mei-Chin Lu, Kuan-Hung Lin, Shih-Chieh Chang.

**Investigation:** Yi-Chun Lai, Mei-Chin Lu, Kuan-Hung Lin.

**Project administration:** Wei-Shu Wang, Su-Shun Lo, Shih-Chieh Chang.

**Resources:** Wei-Shu Wang, Su-Shun Lo.

**Validation:** Jiun-I Lai.

**Writing – original draft:** Yi-Chun Lai, Shih-Chieh Chang.

**Writing – review & editing:** Yi-Chun Lai, Jiun-I Lai, Wei-Shu Wang, Su-Shun Lo, Shih-Chieh Chang.
